# Acetaldehyde, Motivation and Stress: Behavioral Evidence of an Addictive *ménage à trois*

**DOI:** 10.3389/fnbeh.2017.00023

**Published:** 2017-02-09

**Authors:** Anna Brancato, Gianluca Lavanco, Angela Cavallaro, Fulvio Plescia, Carla Cannizzaro

**Affiliations:** Department of Sciences for Health Promotion and Mother and Child Care “G. D’Alessandro”, University of PalermoPalermo, Italy

**Keywords:** acetaldehyde, operant behavior, dopamine, endocannabinoids, stress

## Abstract

Acetaldehyde (ACD) contributes to alcohol’s psychoactive effects through its own rewarding properties. Recent studies shed light on the behavioral correlates of ACD administration and the possible interactions with key neurotransmitters for motivation, reward and stress-related response, such as dopamine and endocannabinoids. This mini review article critically examines ACD psychoactive properties, focusing on behavioral investigations able to unveil ACD motivational effects and their pharmacological modulation *in vivo*. Similarly to alcohol, rats spontaneously drink ACD, whose presence is detected in the brain following chronic self-administration paradigm. ACD motivational properties are demonstrated by operant paradigms tailored to model several drug-related behaviors, such as induction and maintenance of operant self-administration, extinction, relapse and punishment resistance. ACD-related addictive-like behaviors are sensitive to pharmacological manipulations of dopamine and endocannabinoid signaling. Interestingly, the ACD-dopamine-endocannabinoids relationship also contributes to neuroplastic alterations of the NPYergic system, a stress-related peptide critically involved in alcohol abuse. The understanding of the *ménage-a-trois* among ACD, reward- and stress-related circuits holds promising potential for the development of novel pharmacological approaches aimed at reducing alcohol abuse.

## Introduction

It is a matter of fact that the efficacy of current medications for alcohol-related pathological traits remains modest, since the incomplete understanding of the neurobiological background beyond alcohol central effects hampers the development of successful pharmacological therapies (Franck and Jayaram-Lindström, 2013). Alcohol acts at multiple biological targets (Mascia et al., 2001; Martire et al., 2002; Martí-Prats et al., 2013; Zorumski et al., 2014) and its extended use profoundly dysregulates key neurochemical circuits that drive incentive-salience/reward (dopamine, endocannabinoids) and stress-related response (corticotropin-releasing hormone [CRH], Neuropeptide Y [NPY]) within the brain (Koob, 2013). Moreover, the products of alcohol biotransformation, primarily acetaldehyde (ACD), also contribute to its mechanism of action with their own behavioral and neuropharmacological effects (Arizzi et al., 2003; Correa et al., 2003; Pardo et al., 2013; Segovia et al., 2013).

ACD is produced in the human body after the consumption of alcohol in a tissue-specific fashion (Cohen et al., 1980; Ramchandani et al., 2001; Edenberg, 2007), and occurs naturally in alcoholic beverages. Indeed, high ACD concentrations were detected in a number of products, including apple wines and ciders from Germany, France and Scotland, fortified wines and spirits such as sugarcane spirits from Guatemal and Brazil (cuxa; cachaça) (Miranda et al., 2007; Oliveira et al., 2008; Kanteres et al., 2009), agave spirits from Mexico (Lachenmeier et al., 2006), certain spirits from China and calvados from Europe (Lachenmeier and Sohnius, 2008; Linderborg et al., 2008; Lachenmeier et al., 2015). Despite preclinical research has traditionally disregarded the role of taste and post-ingestive influences as independent regulators of motivation to drink alcohol, clinical studies on alcoholism have frequently recognized the significance of alcohol chemosensory stimuli in eliciting craving and associated drug-seeking responses in alcohol-experienced individuals (Stormark et al., 1995; Grüsser et al., 2000). Both alcohol and ACD possess complex chemosensory attributes detected via sensory receptors, which gain immediate access to the central nervous system. Importantly, these sensory pathways are linked to limbic forebrain and cortical areas involved in controlling ingestive motivation and feeding (Kareken et al., 2004; Yamamoto, 2006; Filbey et al., 2008).

Depending on alcohol doses and modalities of administration, peripheral ACD can cross the blood-brain barrier and potentially add to locally formed ACD, produced from alcohol via the brain specific catalase system. On the other hand, increasing evidence shows that ACD is detected in the brain after its oral introduction, when single high systemic concentrations are used, or when chronic exposure occurs (Tabakoff et al., 1976; Heap et al., 1995; Ward et al., 1997; Quertemont et al., 2004; Plescia et al., 2014, 2015a; Jamal et al., 2016).

Whatever its source, either as original substance or as alcohol metabolite, ACD has been largely involved in the mediation of alcohol effect, although its contribution to the development of alcohol abuse still needs to be elucidated.

**Figure 1:**
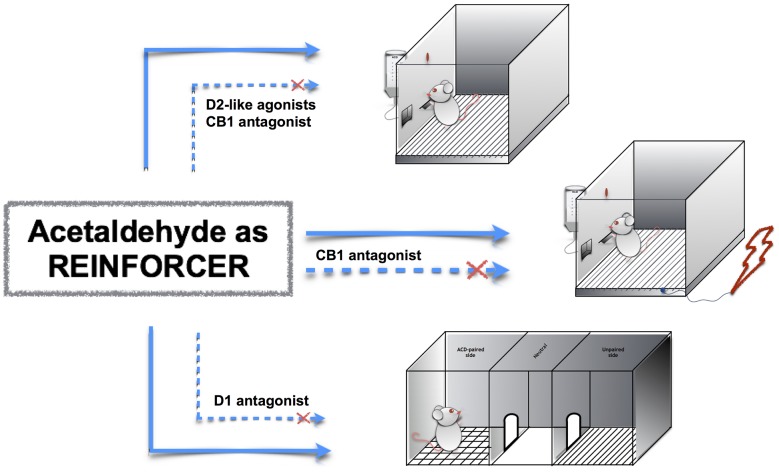
**Acetaldehyde (ACD) motivational properties in operant responding, operant-conflict paradigm and conditioned place preference (CPP).** ACD induces operant responding, operant-conflict behavior and CPP (top to bottom, straight lines); the pharmacological modulation of dopamine and endocannabinoid receptors controls ACD-induced behaviors (dashed lines).

The neuropharmacology of ACD is of particular interest, as ACD interacts both with reward- and stress-related circuits in the brain. For this reason, this mini-review article focuses on ACD motivational properties and examines its interplay with relevant neurotransmitters for motivation-and stress-related response, such as dopamine and endocannabinoids (Figure 1). Indeed, a deeper understanding of ACD neuropharmacology could provide further venue for the development of innovative medication for alcohol use disorders.

## ACD Mirroring Alcohol Effect in the Brain

Looking at the literature, ACD has been investigated primarily as a metabolite raising the idea that it could not only produce aversive reactions in the whole body, but rather may contribute to alcohol mechanism of action in the brain (Plescia et al., 2015b; Cavallaro et al., 2016). Progressively the observation that direct administration of ACD in experimental animals resulted in behavioral effects that are comparable to those induced by alcohol strengthened this concept (Quertemont et al., 2005; Correa et al., 2012). For instance, systemic ACD administrations induced depression in locomotor activity (Myers et al., 1987), impairment of spatial memory (Abe et al., 1999), alcohol-like discrimination (Redila et al., 2002), sedative and hypnotic effects (Quertemont et al., 2004).

Notably, ACD has been implicated in alcohol stimulating effects on the reward pathway in the brain, i.e., ventral tegmental area (VTA) and nucleus accumbens (NAc), that lead to positive reinforcement and mediate alcohol consumption (Brown et al., 1979; Amit and Smith, 1985; Aragon and Amit, 1992; Tampier et al., 1995; Smith et al., 1997). Recently, specific gene-blocking techniques that allow inhibiting catalase in the VTA and thus, the production of ACD from alcohol, demonstrated that ACD mediates alcohol-reinforcing effect in self-administration paradigms. In these studies, microinjection of lentiviral vector encoding anticatalase shRNA into the VTA strongly decreased voluntary alcohol consumption in rats and abolished the increased dopamine release in NAc induced by acute administration of alcohol (Karahanian et al., 2011; Quintanilla et al., 2012). Moreover, VTA anticatalase shRNA injection reduced the marked increase in alcohol intake that follows a period of deprivation, an effect that was proposed to reflect increased reinforcing value of alcohol (Tampier et al., 2013).

If ACD formed from alcohol is responsible for the development of alcohol-related behaviors, then chronic administration of ACD alone should produce behavioral and neurochemical responses of an “addictive” -type.

## ACD as a Reinforcer

ACD’s own reinforcing properties were first shown by conditioned place preference (CPP), behavioral paradigm widely used to explore rewarding effects of drugs. Laboratory rats receiving intracerebroventricular (icv) ACD infusions showed increased preference for environmental cues previously paired with the drug administration (Smith et al., 1984).

A strong preference for ACD-paired environment and stimuli was also observed when ACD administration was either intraperitoneal or oral (Quertemont and De Witte, 2001; Peana et al., 2008).

Place conditioning is suggestive of drug-associated reinforcement, although it may not be clear what exactly the procedure measures. Indeed, it focuses on automatic or implicit expressions of reward, rather than active demonstration of motivated behavior.

Thus the positive reinforcing properties of ACD were more specifically explored by the evaluation of acquisition and maintenance of ACD drinking behavior in self-administration paradigms in rats. Drug self-administration is directly under rat control, and the amount of drug consumed is widely used to infer drug hedonic properties: positively reinforcing drugs will be readily and avidly self-administrated. As alcohol, ACD is voluntary self-administered in two-bottle choice-drinking paradigm and its consummatory behavior was dose-dependent, in that ACD intake increased when higher solution strength was provided (Plescia et al., 2015a; Brancato et al., 2016b). The flavor and taste of ACD solution have been proposed to take part to the reinforcing properties and may actually serve as conditioned stimuli of post-ingestional effects (Cannizzaro et al., 2011).

Little is known on the molecular targets that account for ACD complex flavor. However, ACD directly activates the sensory neuronal TRP channels TRPA1 that are relevant for taste and chemesthesis (Bang et al., 2007; Roper, 2014). With chronic exposure, sensory and post-ingestive inputs become intimately integrated, such that these stimuli gain meaning for the addicted organism (Brasser et al., 2015). Natural ACD self-administration provides a framework for moving beyond the dissociation between the sensory and post-absorptive effects of ACD to the understanding of their neurobiological integration and significance for sensory processing of alcoholic beverages and alcohol addiction.

The suggestion that ACD may be endowed with positive reinforcing properties was further investigated by using a variety of operant self-administration paradigms.

The operant self-administration is a commonly used model in which animals are trained to emit a specific response (lever press or nose poke) for gaining the reinforcement (Samson et al., 1988). Operant behavior for ACD was readily acquired by rats, both through icv and intravenous routes of administration (Brown et al., 1980; Myers et al., 1984). In details, Rodd et al. (2003, 2005) demonstrated that rats selectively bred as alcohol drinkers self-administered both alcohol and ACD directly into the VTA, where ACD showed reinforcing effects at concentrations 1000 lower than those required for alcohol. Unselected animals also perform lever pressing for obtaining ACD through the natural oral route. Indeed ACD was reported to induce and maintain operant drinking behavior according to fixed and progressive ratios of reinforcement (Peana et al., 2011; Cacace et al., 2012). Apart from drug taking, the operant conditioning paradigm serves as an invaluable tool in addiction research, since it enables researchers to explore discrete features of addictive behavior, as reported for humans in the DSM-V (American Psychiatric Association, 2013). Indeed, different schedules of drug reinforcement critically model distinct aspects of incentive motivation for the drug, such as drug seeking and relapse following periods of abstinence, and maintained alcohol use despite adverse consequences that constitute central issues of the translational research on addiction. The employment of such tailored paradigms showed that ACD acts as positive reinforcement that elicits challenging behavior, such as craving and relapse, as shown for alcohol. Indeed, ACD-drinking rats displayed resistance to extinction i.e., the emission of high number of operant responses when reinforce delivery was withheld- and a powerful deprivation-effect when ACD availability was resumed after repeated cycles of deprivation (Peana et al., 2010; Cacace et al., 2012; Plescia et al., 2013; Brancato et al., 2014). The motivational properties of ACD have been further measured by the operant-conflict paradigm, where an aversive stimulus is associated with rat operant response for ACD. Indeed, when a mild foot-shock was delivered following each lever press rewarded with ACD (punished response), ACD-drinking rats were not discouraged from lever pressing and emitted higher number of punished responses than control rats (Cacace et al., 2012). In the Geiller-Seifter procedure, anxiolytic drugs do not affect the unpunished component of operant responses, whereas drugs with non-specific motor effects decrease it. Actually, ACD was able to increase the unpunished responses, although to a lesser extent than the punished ones, suggesting a prevailing motivational effect, rather than anti-conflict properties (Cannizzaro et al., 2011; Cacace et al., 2012).

## ACD and Stress Response

A large and consistent body of literature, on the other hand, shows that both acute and chronic ACD peripheral administrations were associated with anxiety-like behavior in the elevated plus maze (Correa et al., 2005; Plescia et al., 2015a) and with the recruitment of peripheral and central stress response. In particular, ACD was shown to mediate alcohol-induced activation of the hypothalamic-pituitary-adrenal (HPA) axis, since ACD increased plasma corticosterone levels (Kinoshita et al., 2001; Escrig et al., 2012) and induced the release of CRH in a dose-dependent manner (Cannizzaro et al., 2010). Notably, when the oxidation of alcohol into ACD by catalase was inhibited by 3-amino-1,2,4-triazole, CRH release in the presence of alcohol was prevented. Furthermore, the administration of D-penicillamine, an ACD-trapping agent, inhibited ACD- induced CRH release, demonstrating that ACD is the primary mediator of alcohol activity on the HPA axis (Cannizzaro et al., 2010).

The stress system contributes to various extents to the development of alcohol-related behaviors.

Indeed, alcohol- and ACD induce an activation of the stress system that can facilitate behavioral reactivity in aversive conditions (Cacace et al., 2011, 2012; Plescia et al., 2015a). On the other hand repeated cycles of alcohol and ACD intoxication deeply affect the homeostasis of brain stress- and anti-stress system. Indeed, both chronic alcohol and ACD excessive consumption decreased the expression of the anxiolytic peptide NPY in limbic brain regions, such as hippocampus and ventral striatum (Kinoshita et al., 2000; Olling et al., 2007; Plescia et al., 2014). Most importantly, the discontinuation of chronic and high doses of ACD induced a constellation of behavioral signs, such as general hyperactivity, irritability, tail tremors, tail stiffness, general tremor and spasticity, which recall alcohol withdrawal syndrome. ACD withdrawal signs exhibited minor severity, were observed at 12 h from the last ACD administration, started to decline at 16 h and disappeared at 36 h abstinence (Plescia et al., 2014). It is worth noting that during this time CRH expression increased and NPY expression levels decreased in limbic brain areas and in the hypothalamus, causing the occurrence of the aversive psychological state characteristic of withdrawal. These modifications are consistent with the so-called involvement of the “dark side”, or stress systems, in the development of alcohol use problems and abuse vulnerability (Thiele et al., 1998; Koob, 2013; Barkley-Levenson et al., 2016). Individuals would consume alcohol in an attempt to return to homeostasis via a negative reinforcement process that maintains and promotes drug taking. Similarly to alcohol, ACD would contribute to engender an aversive (anxious, depressive) state by bidirectional effects on the two major and functionally opposite stress-related peptides, CRH and NPY, thus perpetuating excessive alcohol consumption.

## Pharmacological Characterization of ACD-Related Behavior

### ACD and Dopamine

Although the mechanisms by which ACD elicits its effects are poorly explored, this compound activates the neuronal firing of dopaminergic neurons in the VTA, an effect that is mediated by salsolinol, the condensation product of ACD and dopamine (Melis et al., 2007, 2015). Importantly, ACD elicits dopamine release in the NAc shell (Foddai et al., 2004; Melis et al., 2007; Enrico et al., 2009) at the same doses used in CPP studies (Peana et al., 2008, 2009; Spina et al., 2010).

This is not surprising, since the acquisition of drug-induced CPP is critically controlled by dopamine transmission and D1 receptors in the NAc shell (Di Chiara et al., 2004; Tzschentke, 2007). Spina et al. (2010) demonstrated that this also applies to ACD. The blockade of D1 receptors during ACD conditioning, through the pre-treatment with SCH 39166, a D1 dopamine receptor antagonist, also prevented the acquisition of CPP for ACD. In this regard, interference not only on incentive learning processes but also on dopamine-mediated reward has been proposed (Di Chiara et al., 2004).

Besides, dopamine plays a fundamental role in the expression of operant behavior elicited by rewards and reward-related stimuli. Release of dopamine in the NAc shell by Pavlovian stimuli induces an appetitive state of incentive arousal (state—hedonia, euphoria) that facilitates the rate of current instrumental behavior, the acquisition and expression of secondary reinforcement, as well as the consolidation of mnemonic traces of salient stimuli associated with affective states.

It is proven that ACD stimulates dopamine release, and that dopamine increase in the limbic regions accounts for addictive behavioral traits in the rat. Thus, modulating dopamine release or deactivating dopamine signaling could represent a tool able to interrupt the addictive cycle. Indeed, the involvement of dopamine transmission in ACD-related operant behavior was explored by the administration of a D2 dopamine receptor agonist, quinpirole, which at low doses preferentially activates presynaptic D2 dopamine autoreceptors. Thus, by functionally reducing ACD-induced dopamine release, quinpirole decreased the number of lever presses for ACD, also during extinction and, after ACD deprivation, during relapse (Rodd et al., 2005; Brancato et al., 2014). Quinpirole was then able to restrain dopamine signal as supporter of the incentive and rewarding properties of ACD, which indeed was less demanded by the rats. Interestingly, in accordance with chronic alcohol-induced down regulation of dopamine signaling in the limbic regions, chronic ACD could exert a profound disarrangement in dopamine output to the NAc during withdrawal (Rossetti et al., 1992). Indeed, when ropinirole was sub-chronically administered during ACD deprivation, a decrease in operant responses and ACD intake was observed during relapse (Brancato et al., 2014). This post-synaptic D2 dopamine receptor agonist, useful to restore dopaminergic tone in Parkinson’s disease (Tel et al., 2002), likely produced a stimulation of dopamine D2 signaling able to turn off rats craving when ACD was available. This evidence contributes to the suggestion that ACD interaction with the dopamine system plays a role in the development of discrete features of addictive behavior that can be especially relevant to alcohol use disorders.

### ACD and Cannabinoids

Along with the dopaminergic transmission, the endocannabinoid system plays an important role in value attribution processing and in modulation of drug-seeking behavior (Serrano and Parsons, 2011; Brancato et al., 2016a; Henderson-Redmond et al., 2016), in view of its role as fine modulator of incoming inputs within the limbic brain regions (D’Amico et al., 2004; Cannizzaro et al., 2006; Melis et al., 2012). Indeed, in rodents, treatment with the CB1 receptor inverse agonist SR141716A (Rimonabant), or CB1 genetic deletion, lead to a reduction in alcohol operant drinking and a decrease in stress-induced alcohol relapse, whereas cannabinoid antagonists mitigate alcohol withdrawal symptoms (Kleczkowska et al., 2016).

Consistently with this significant background, the systemic administration of the selective CB1 receptor antagonist AM281 was evaluated on the operant behavior for ACD. In details, CB1 receptor blockage decreased ACD-seeking behavior during extinction and decreased ACD lever pressing and intake following forced abstinence. Most importantly, the CB1 antagonist decreased the punishment resistance observed in ACD-drinking rats in the operant-conflict paradigm, when the foot-shock was associated with ACD delivery (Plescia et al., 2013). These data suggest that the reinforcing properties of ACD involve endocannabinoids production, which in turn, modulate dopamine mesocorticolimbic pathway and stress response through CB1 receptors. Indeed a recent research employing a binge-like drinking paradigm, pointed to the endocannabinoids as mediators of the detrimental effects exerted by ACD chronic consumption and withdrawal on neuropeptidergic homeostasis, and in particular on the expression of the anti-stress NPY (Plescia et al., 2014). In this study, the administration of the CB1 receptor antagonist was able to ameliorate the behavioral signs that followed withdrawal from chronic ACD; this effect was accompanied by a time- and region-dependent increase in the number of NPY-positive neurons both in the hippocampus and in the NAc. These data prompted us to speculate that ACD binge-like treatment might increase the production of endocannabinoids, thus resulting in downregulation of NPY expression in the hippocampus and in the NAc. Accordingly, during early and prolonged ACD withdrawal, endocannabinoids production may decrease while NPY expression progressively rises. This return to homeostasis can likely contribute to controlling neuronal hyperexcitability and the related behavioral signs (Plescia et al., 2014). Hence, the pharmacological inhibition of CB1 signaling represents a promising strategy for counteracting the neurochemical imbalance associated with ACD- and alcohol-withdrawal syndrome.

## Conclusions

A deeper understanding of the “*ménage à trois*” between ACD, reward- and stress systems is crucial to untangle the etiology of alcohol-related behaviors. Increasing attention must be paid to alcohol, and indirectly to ACD, ingestion during gestation and lactation since the neuronal systems suffer from a severe vulnerability (Cannizzaro et al., 2002, 2005), and ACD has not been studied in the perinatal period yet.

The pharmacological targeting of the endocannabinoid system can exert profound influence on the positive and negative reinforcing effects of ACD, and might accelerate the development of more effective therapeutic interventions to reduce the incidence of alcohol abuse and alcoholism.

## Author Contributions

AB, GL, AC, FP and CC wrote major parts of the article. All authors critically reviewed and edited the article. The review article was written based on the expertise of the authors, who have sourced the article on PubMed and Google Scholar.

## Funding

This study was supported by research grant 2012-ATE-0167 provided by the University of Palermo, Italy.

## Conflict of Interest Statement

The authors declare that the research was conducted in the absence of any commercial or financial relationships that could be construed as a potential conflict of interest.
